# In Silico Analysis Highlights Potential Predictive Indicators Associated with Secondary Progressive Multiple Sclerosis

**DOI:** 10.3390/ijms25063374

**Published:** 2024-03-16

**Authors:** Marco Calabrò, Maria Lui, Emanuela Mazzon, Simone D’Angiolini

**Affiliations:** IRCCS Centro Neurolesi “Bonino-Pulejo”, Via Provinciale Palermo, Contrada Casazza, 98124 Messina, Italy; marco.calabro@irccsme.it (M.C.); maria.lui@irccsme.it (M.L.);

**Keywords:** secondary progressive multiple sclerosis, transcriptomic analysis, blood samples, brain samples, biomarkers

## Abstract

Multiple sclerosis (MS) is a complex inflammatory disease affecting the central nervous system. Most commonly, it begins with recurrent symptoms followed by partial or complete recovery, known as relapsing–remitting MS (RRMS). Over time, many RRMS patients progress to secondary progressive MS (SPMS), marked by gradual symptom deterioration. The factors triggering this transition remain unknown, lacking predictive biomarkers. This study aims to identify blood biomarkers specific to SPMS. We analyzed six datasets of SPMS and RRMS patients’ blood and brain tissues, and compared the differential expressed genes (DEGs) obtained to highlight DEGs reflecting alterations occurring in both brain and blood tissues and the potential biological processes involved. We observed a total of 38 DEGs up-regulated in both blood and brain tissues, and their interaction network was evaluated through network analysis. Among the aforementioned DEGs, 21 may be directly involved with SPMS transition. Further, we highlighted three biological processes, including the calcineurin–NFAT pathway, related to this transition. The investigated DEGs may serve as a promising means to monitor the transition from RRMS to SPMS, which is still elusive. Given that they can also be sourced from blood samples, this approach could offer a relatively rapid and convenient method for monitoring MS and facilitating expedited assessments.

## 1. Introduction

Multiple sclerosis (MS) is a complex inflammatory demyelinating disease that affects the central nervous system (CNS) [1]. Its origin is believed to result from intricate interactions between genetic and environmental factors, although the full details of these dynamics remain elusive [2]. Beyond its inflammatory characteristics, MS is also widely recognized as a neurodegenerative condition [3]. MS is a condition that exhibits a low incidence during childhood but undergoes a notable increase after the age of 18. Its prevalence peaks between the ages of 20 and 40 years, with the average onset occurring around 30 years, and women typically experience symptoms 2–5 years earlier than men [4]. In Europe, MS occurs at a rate of approximately 83 cases per 100,000 individuals, and the mean annual incidence rate hovers around 4.3 cases per 100,000 [5]. Individuals diagnosed with MS face a reduced life expectancy, with a shortening of their life span by 7–10 years. Although the standardized mortality ratio has tripled, there has been discernible improvement in this ratio over the last few decades [6]. Despite advancements in medical understanding, the primary trigger for the immune response in MS remains unidentified. In the initial phases of the inflammatory cascade, a reaction is triggered against myelin antigens, encompassing the myelin basic protein, proteolipid protein, myelin/oligodendrocyte glycoproteins, and gangliosides. The lesions associated with MS are classified as acute, chronically active, and inactive. While lesions may manifest throughout the CNS, they are most frequently observed in the optic nerves, cerebral periventricular white matter, brainstem, and white-matter tracts of the spinal cord [7]. The course of MS exhibits considerable variability; however, in the majority of cases, it entails the onset of recurrent clinical symptoms followed by either complete or partial recovery, a characteristic feature of the classic relapsing–remitting form of MS (RRMS). However, approximately 15% of individuals with MS experience relentless disease progression from the onset, known as primary progressive MS (PPMS) [8]. After 10–15 years of the disease, nearly 50% of untreated RRMS patients enter a progressive phase characterized by a slow and continuous deterioration of clinical symptoms over many years. This stage is termed secondary progressive multiple sclerosis (SPMS) [9]. Nevertheless, studies have demonstrated that frequent relapses within the initial 2 years are correlated with later disability, increasing the likelihood of developing SPMS and shortening the latency of its onset [10]. Identifying the precise point of the transition to SPMS can be challenging and is often acknowledged only retrospectively, sometimes years after subtle signs of progression first emerge [11]. The investigation into MS remains dynamic, as many aspects of its intricate pathogenesis remain unknown, leading to a lack of curative medications for affected individuals. Ongoing research aims to explore potential imaging and laboratory biomarkers that can differentiate SPMS from RRMS, characterize the transition from RRMS to SPMS, and potentially predict this transition. While various therapies designed to impact the immune response against the CNS are in use, they primarily serve to slow down and/or mitigate the severity of the clinical course of MS. This sobering reality underscores the significance of exploring other components in the pathology of MS [12]. The use of transcriptomics data may provide helpful insights to better define MS etiopathogenesis [13]. To better elucidate the specific cellular mechanisms underlying SPMS compared to RRMS, and thus enable early identification of MS subtypes, we analyzed brain and blood expression datasets for SPMS compared to a group of healthy controls (HCs). Peripheral blood biomarkers furnish crucial insights into both the immune triggers of MS and the therapeutic efficacy of administered drugs [14]. Notably, the accessibility of blood surpasses that of other bodily tissues. Additionally, blood serves as a conduit for molecules originating from diverse tissues, offering a comprehensive reflection of the overall biological status of the body [15]. The aim of this study was to pinpoint DEGs that could contribute to a deeper comprehension of the transition from RRMS to SPMS. Additionally, we directed our investigation towards elucidating the significance of altered genes within cells, aiming to clarify their role in altered cellular mechanisms.

## 2. Results

In the present study, we analyzed MS databases containing information on gene expression data from blood and brain tissues of patients with SPMS and HCs. Our main focus was to provide useful information that may enable the identification of potential diagnostic, predictive, or treatment response biomarkers, as well as key biological pathways involved in the SPMS transition.

### 2.1. Differential Expression Analysis (DEA) of SPMS Data

**Figure 1:**
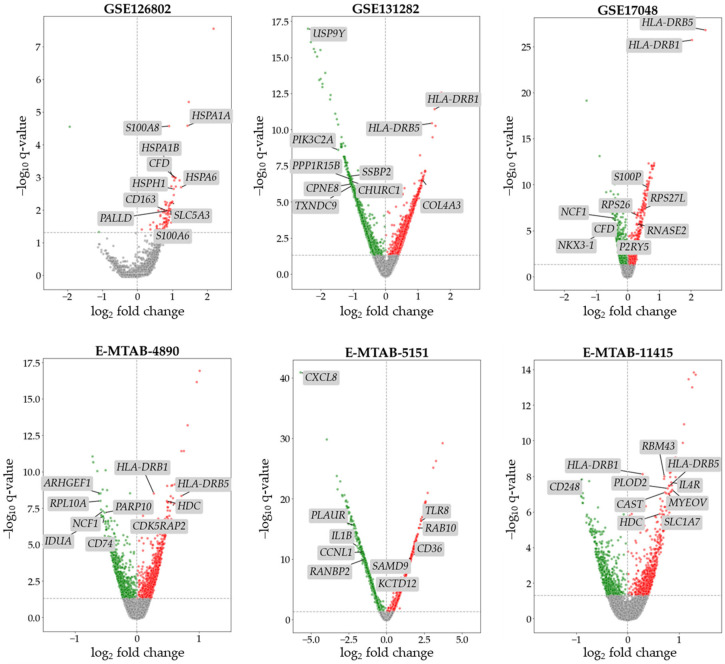
In the dot plot, we report all of the genes explored in the DEA for each dataset. The line that intercepts the y-axis is related to our threshold of significance for the q-values (0.05); all of the genes above this line are considered to be differentially expressed. On the x-axis, we report the log_2_ fold change that discriminates up- and down-regulated DEGs. DEGs resulted significantly up-regulated are highlighted in red, while DEG significantly down-regulated are highlighted in green. Genes not differentially expressed are reported in gray. Labels reports the top 10 significant DEGs that survived the filtering step (please refer to Section 2.3).

The data collected from each dataset on SPMS patients and HCs were used to perform DEA, which constituted the first step of our analysis. This analysis aimed to provide a preliminary pool of DEGs to be further analyzed in the study’s following steps. We utilized the rank product method, through the BioTEA tool, to diminish biases related to the use of different samples/arrays. In the DEA, we highlighted a set of DEGs for each dataset. The results of the analysis are shown in Figure 1, where volcano plots summarize the distribution of up-regulated, down-regulated, and non-significant altered genes for each dataset. Specific details on the number, distribution, and names of the DEGs are reported in Supplementary Table S1.

As can be observed in Figure 1, the DEA returned heterogenous sets of DEGs. Interestingly, most of the top-ranking DEGs in each dataset were not replicated in other datasets, further stressing the heterogeneity and complexity of MS in general and the SPMS subtype in this specific case. As such, a filtering step was deemed necessary to select the DEGs shared among multiple datasets.

### 2.2. DEG Filtering and Selection

To identify the DEGs reflecting alterations occurring in both brain and blood tissues, we conducted a comparative analysis across the results obtained from the previous step. Specifically, we selected DEGs present in at least 50% of the brain datasets that were also identified as DEGs in the blood datasets with consistent expression patterns across at least 75% of the datasets. This filtering process yielded a set of 469 brain DEGs that exhibited differential expression in both brain and blood tissues, thereby representing potential biomarker candidates for the condition under study. The top 10 DEGs that survived the filtering step for each dataset are reported in Figure 1. From the filtered pool of DEGs, we further refined our selection by focusing on those that were up-regulated in both brain and blood tissues, resulting in a subset of 100 DEGs (referred to as RuLu). By focusing on up-regulated DEGs, we aimed to streamline the identification process and prioritize candidates with the potential to be used as robust biomarkers. This approach not only increased the likelihood of identifying clinically relevant markers, but also facilitated the translation of our research findings into practical diagnostic or therapeutic applications. Therefore, the selection of up-regulated DEGs aligned with the overarching objective of identifying actionable biomarkers with translational significance. Supplementary Figure S1 shows the number of DEGs that survived the filtering step, and Supplementary Table S2 provides the details concerning these genes.

### 2.3. Network Analysis

**Figure 2:**
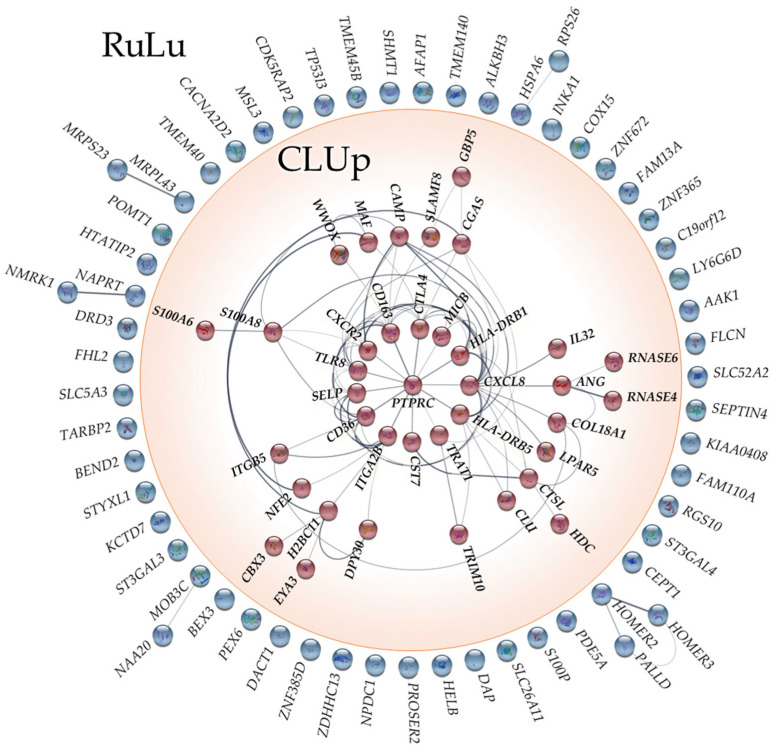
Network composed of the 100 DEGs related to RuLu. The subnetwork composed of the 38 CLUp DEGs is highlighted in red. All of these DEGs were found to be up-regulated in both brain and blood tissues of SPMS patients.

The 100 identified Rulu DEGs were used as the input for a network analysis, utilizing the STRING plugin v2.0.2 within Cytoscape software v3.10.1. This analysis was performed with the aim of exploring and assessing potential interactions among the DEGs’ encoded proteins, to ultimately elucidate the interconnectedness of these proteins and evaluate their collective role in biological processes. The resulting network revealed a distinct subcluster, denoted as CLUp, comprising 38 DEGs. These 38 interconnected elements highlight the presence of potentially coordinated biological processes or pathways that may be perturbed in the context of the condition under investigation. Figure 2 illustrates the findings of the network analysis, providing valuable insights into the interconnectedness of the identified DEGs and their potential implications for understanding disease mechanisms.

### 2.4. Ontology Enrichment

**Figure 3:**
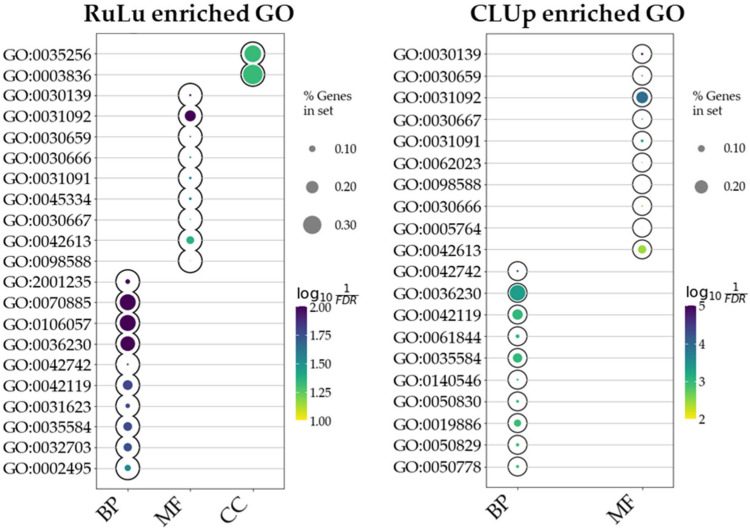
This plot shows the enriched ontologies resulting from the ORA for the RuLu and CLUp DEGs, respectively. The size of the bubble represents the ratio of DEGs divided by the total number of genes composing each ontology term (in %). The hue represents the significance scores (−log_10_ FDR). The x-axis indicates the ontologies used: biological processes (BPs), cellular components (CCs), and molecular functions (MFs).

To elucidate the predominant gene ontologies (GOs) characterizing our identified DEGs, we conducted a comprehensive over-representation analysis (ORA) encompassing all selected DEGs as well as the cluster derived from the network analysis. This analysis used information on biological process (BP), cellular component (CC), and molecular function (MF) ontologies to highlight significant over-represented ontologies, providing a broader perspective on potential dysregulations across these various ontological categories. The ORA conducted using all 100 DEGs from the RuLu group revealed enrichment in 29 BP, 9 CC, and 2 MF ontologies. Similarly, the ORA performed on the CLUp DEGs exhibited enrichment in 95 BP, 37 CC, and no MF ontologies. It is noteworthy that in both cases, the enriched ontologies were determined based on the significance of the q-values, highlighting those with the most statistically significant associations. The top 10 enriched ontologies for RuLu and CLUp are presented in Figure 3. These selections were made based on the lowest q-values, signifying the highest degree of statistical significance. Furthermore, Supplementary Table S3 provides a comprehensive list of all enriched GO terms along with their associated DEGs.

**Figure 4:**
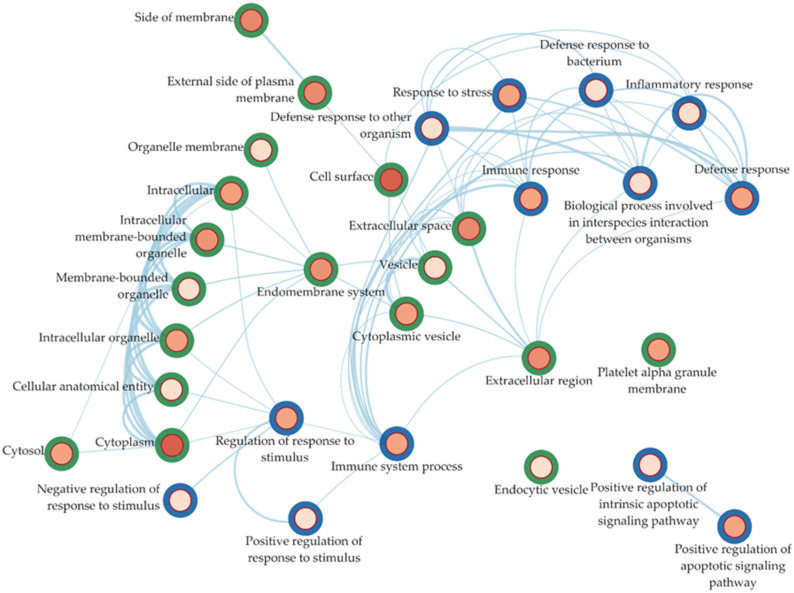
This figure highlights the interactions between the enriched ontologies for the DEGs belonging to the RuLu group. The BP ontologies are outlined in blue, while the CC ontologies are outlined in green. The hue represents the significance of the nodes, with red being more significant. Ontologies with a high number of shared genes were automatically fused together.

To comprehensively observe how the enriched ontologies interact with each other, we also built an interaction network of the ontologies, aiming to provide insights into the interplay and potential crosstalk among various biological pathways and cellular components implicated in the condition under investigation. The functional analysis indicated immune processes as the predominant pathways associated with SPMS, consistent with the existing literature and supported by the original authors of the investigated datasets [16,17,18,19,20,21]. These DEGs primarily localized on cell surfaces, emphasizing the involvement of immune receptors in MS pathology. In Figure 4, we report the BP and CC interaction network.

### 2.5. Comparison with RRMS Patients’ Data

Up to this step, the analyses performed provided information on the DEGs likely involved with the SPMS phenotype and the processes potentially altered in this condition regardless of factors such as the sample origin, as they resulted from the intersection of multiple datasets.

**Figure 5:**
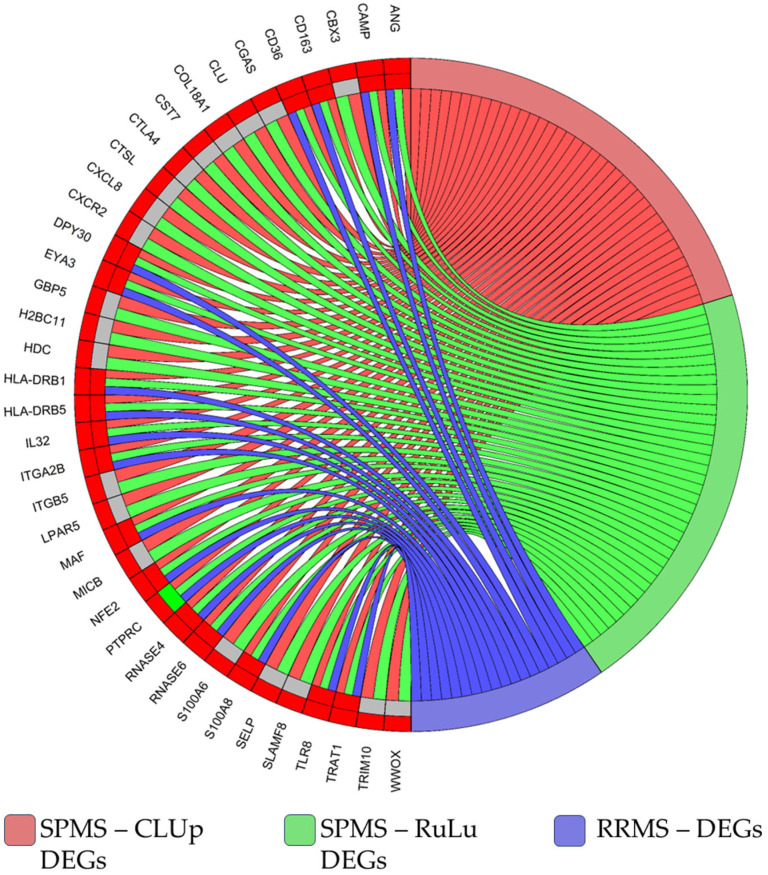
In this chord plot, we report the CLUp DEGs (left-hand side) and the phenotypic groups in which they were differentially expressed (right-hand side). On the left, the outer ring reports the expression status in SPMS patients, while the inner one reports the expression status in RRMS patients. Red genes are up-regulated and green are down-regulated, while gray represents no differential expression. DEGs belonging to the CLUp group are linked to the red sector on the right-hand side; DEGs belonging to the RuLu group are linked to the green sector; and genes found to also be differentially expressed in RRMS patients are linked to the blue sector. The 21 selected genes were up-regulated in SPMS (outer ring in red) and down-regulated or not DEGs in RRMS (inner ring in gray or green).

However, the CLUp DEGs may also be indicators of MS in general, since they may also be dysregulated in other subtypes. To assess their potential use as biomarkers of progression/transition from RRMS to SPMS, we needed to prune from our DEGs all of the elements that show similar dysregulation in the RRMS subtype. To perform such pruning, we performed a DEA on the patients with RRMS vs. HCs of the blood dataset. The selection of the blood dataset was based on the fact that eventual biomarkers should be present in blood (for easiness of use); any other element which also results in DEGs in RRMS patients would not be useful as a progression biomarker. The execution of the DEA and the subsequent filtering followed the same scheme used for the SPMS data analysis (please refer to Section 2.1 and 2.2). As expected, the results obtained from the RRMS DEA evidenced a partial overlap of DEGs. In detail, for RuLu, we found 37 DEGs with the same expression behavior; 4 DEGs that appeared in both SPMS and RRMS but with opposite expression behavior; and 67 genes specific for SPMS. Finally, for CLUp, we found 17 genes with the same expression behavior, which we deemed not eligible as biomarkers specific for SPMS; 1 DEG that appeared in both SPMS and RRMS but with opposite expression behavior; and 20 genes specific for SPMS. These latter 21 genes represent the DEGs that more likely have the potential to be biomarkers of the RRMS transition to SPMS. The results are summarized in Figure 5 for the CLUp DEGs and in Supplementary Figure S2 for the RuLu DEGs. Further details on the RRMS DEA are reported in Supplementary Table S4.

As an additional step, we performed an ORA on the DEG subgroups specific to SPMS and common in RRMS and SPMS to identify BPs specific to SPMS. This analysis was performed with the aim of identifying potential processes whose alteration may constitute a potential mechanic behind MS’s transition from RRMS to SPMS. To increase the specificity of this comparison, we performed a three-layered assessment. In the first layer, we compared CLUp DEGs that were specific for SPMS vs. DEGs shared with RRMS patients. In the second-layer, we extended the analysis to all DEGs from the RuLu group. In the third layer, we compared all of the SPMS DEGs that exhibited opposite/different behavior in RRMS patients (i.e., up-regulated in SPMS and down-regulated/not DEGs in RRMS) vs. DEGs with the same behavior in both SPMS and RRMS patients. From these comparisons, we selected the BPs that were specifically enriched in SPMS patients but not in RRMS patients. The final results of these analyses are summarized in Table 1, and are detailed in Supplementary Table S5, respectively.

## 3. Discussion

MS exhibits a multifactorial nature, complicating the identification of its genetic background and prediction of its progression across subtypes.

For our study, we used a subset of subjects from six different databases containing information on gene expression from both brain (GSE126802 [16] and GSE131282 [17]) and blood (GSE17048 [18], E-MTAB-11415 [19], E-MTAB-4890 [20], and E-MTAB-5151 [21]) tissues. We opted to use both brain and blood tissues for our analysis. The rationale for this choice was based on the fact that (1) MS is a pathology mainly acting on the CNS [1], and (2) biomarkers should be easily accessible. Thus, we first selected all DEGs from the brain tissues, which should be informative of MS-related expression alterations, and then focused on the ones that also appeared as DEGs in the blood, which is an easily accessible tissue. These choices allowed our analysis to have some advantages: the use of multiple heterogenous samples would highlight alterations caused by the phenotype under investigation, filtering out genes whose dysregulation was due to other – sample-related – factors; the filtering of brain DEGs in the blood helped us to isolate DEGs that were likely involved in MS physiopathology in CNS, and, at the same time, easy to detect given their presence in blood; and the use of both brain and blood samples gave us an increased sample size.

Overall, the results we obtained, especially in first steps of our analyses, are supportive of the important role of immune/inflammatory processes in MS, which constituted the main findings of the original works related to the datasets used [16,17,18,19,20,21], as many of the top genes found to be up-regulated (please refer to Figure 1 and the Supplementary Materials) were related to such processes. The datasets’ original studies also greatly described the shared molecular pathways at work in MS pathogenesis, regardless of the subtype, and were informative of the regulatory architecture behind MS-associated altered gene expression. Different from the datasets’ original works, this study aimed to highlight gene expression alterations that could serve as potential biomarkers for the progression of MS towards the SPMS subtype. Additionally, we sought to infer specific biological processes contributing to this transition: we compared CLUp DEGs with those obtained from RRMS patients versus HCs in blood analyses. Filtering out the common elements, we obtained a set of 21 (up-regulated) DEGs specific for SPMS. This list included *CBX3*, *CGAS*, *CLU*, *COL18A1*, *CST7*, *CTLA4*, *CTSL*, *CXCL8*, *CXCR2*, *GBP5*, *H2BC11*, *HDC*, *ITGB5*, *LPAR5*, *MICB*, *PTPRC*, *S100A6*, *SELP*, *SLAMF8*, *TRIM10*, *WWOX*. The potential role of these genes in MS is discussed as follows.

The *CBX3*-encoded protein has been implicated in T-cell self-tolerance modulation and homeostasis mechanics [22], processes involved in MS. Up-regulation of *CBX3* may lead to consistent inhibition of regulatory CD4^+^ T-cells, diminishing their function as suppressors of autoimmune events. Additionally, CBX3 acts as a positive regulator of the high-affinity antibody response [23,24]. While high-affinity antibody production is crucial for pathogen clearance, it promotes the production of auto-antibodies in MS and other autoimmune diseases, exacerbating immune system effects on patients.

The *CGAS*-encoded protein is involved with immune processes, as well as autoinflammatory and neurodegenerative disorders [25,26]. CGAS produces cyclic GMP-AMP, a second messenger involved in interferon (IFN) production [27]. While IFN has anti-inflammatory properties [27], it has been observed that subjects with autoimmune diseases have an increased/chronic production of IFN and are more prone to produce auto-antibodies [28]. Based on these data, the up-regulation of *CGAS* may be an indicator of chronic inflammation and increased auto-antibody production in MS.

The *CLU* gene encodes for a chaperone promoted as a potential biomarker of MS by independent studies [29]. In certain stress-associated conditions, including active inflammatory states [30], CLU can accumulate in the cytoplasm, where it inhibits apoptosis [31], or in the nucleus, where it promotes apoptosis [32,33]. It can be hypothesized that extended exposure to stressing conditions may lead to intracellular accumulation of CLU in MS, which could trigger pro-apoptotic processes, consequently aggravating the disease.

The *COL18A1* gene encodes for the precursor of endostatin [34], and is up-regulated in several immune-related diseases [35,36]. Although angiogenesis appears to have a deleterious role in MS, this is true only in the early phases of lesion formation [37,38]. In the later phases, this process acquires a beneficial role and contributes to remission after relapses [34]. Further on in MS’s progression, lesions are characterized by a marked hypoperfusion, causing hypoxia and triggering disease progression [34]. According to this data, *COL18A1* may be an indicator of the transition to the advanced phases of MS and, as suggested by our results, it may be a good indicator of the RRMS-to-SPMS transition.

The *CST7* gene is mainly expressed in immune system cells [39], and seems to be linked to demyelination processes. Data from the literature report that *CST7* expression appears to be up-regulated in activated microglia during demyelination, and totally absent in normal brains [40]. This observation highlights the potential of this gene for use as a marker of active demyelination. Notably, CST7 showed a high affinity for cathepsin L, a protein encoded by *CTSL*, another overexpressed gene in our results [41], suggesting a complex balancing mechanism acting between the two elements.

The *CTLA4* gene is involved in T-cell homeostasis and self-tolerance [42]. Despite autoimmunity being linked to its loss [43], our results indicate an up-regulation of *CTLA4*. This overexpression may represent an effort to modulate the immune response, as increased *CTLA4* expression is physiological in activated T-cells [44]. In MS, T-cells’ increased tolerance to this regulatory mechanism [45] may hinder CTLA4’s ability to deactivate/regulate T-cells effectively. The literature suggests that the activation of T-cells in MS is independent of co-stimulatory signals [46], and CTLA4 signaling is impaired in MS patients [42]. Notably, *CTLA4* is down-regulated in RRMS patients [47]. Thus, *CTLA4* overexpression, combined with persistent inflammation, could serve as a potential marker for T-cell regulation loss, particularly prominent in SPMS compared to RRMS.

The *CTSL* gene encodes for an enzyme involved in the cleavage of complement C3 into its active fragments C3a and C3b [48]. C3a promotes T-cell survival and regulates T-cell responses and cytokine production [49,50]. Up-regulation of *CTSL* suggests increased production of C3a fragments. Interestingly, T-cells from patients with autoimmune diseases exhibit CTSL-dependent complement hyper-activation and IFNγ production [48]. This phenomenon may be prominent in the SPMS subtype, due to the chronic inflammatory state. Notably, as previously discussed, this up-regulation may be countered by CST7. However, they usually do not colocalize [51], potentially limiting the inhibition’s effectiveness.

The *CXCL8* gene encodes for a pro-inflammatory chemokine [52,53] mainly involved in recruiting immune cells from the blood and in their adhesion to endothelium [54]. Studies in the literature report an up-regulation of the *CXCL8* receptor in immune cells at the chronic lesions [55], suggesting its potential for use as a biomarker, and, according to our results, it may be specific for SPMS. Notably, *CXCL8* expression is regulated by the calcineurin–NFAT signaling pathway, which was found to be enriched in SPMS subjects [56].

The *CXCR2* gene encodes for the CXCL8 receptor. CXCR2 is an important activator of the immune response and has a key role in immune cells recruitment [57]. This receptor acts in concert with its ligand in MS to promote the heightened inflammatory status [55]. As described before, *CXCR2* appears to be up-regulated in many immune cells, including macrophages, astrocytes, microglia, and oligodendrocytes, in MS lesions [55]. Also, *CXCR2* mRNA levels were found to be higher in brain autopsies from MS subjects [58]. For these reasons, this element may also be a potential marker of MS, especially in the later progressive phases, where a continuous up-regulation is expected.

The *GBP5* gene is involved in cellular inflammatory responses and cytokine release [59,60]. While not directly linked to MS, the literature suggests that GBP5 has a role in selective NLRP3 inflammasome activation [61], a process potentially linked with MS. NLRP3 activation significantly increases neutrophil extracellular trap (NET) formation in the brain [62] with a concomitant increase in the expression of pro-inflammatory receptors such as CXCR2. NET has been implicated in the pathology of MS [63] and disease severity [62]. Thus, GBP5 may be implicated in MS through the activation of NLRP3, consequently acting together with pro-inflammatory elements like CXCL8 and CXCR2, to promote immune cell recruitment and NET formation. Elevated *GBP5* expression may be indicative of this progression, suggesting its potential as a biomarker.

The *H2BC11* gene encodes for a core component of the nucleosome, with no proven association with MS. Data from the literature report that its expression is enriched in immune cells, especially neutrophils [64], and its potential role as an indicator of immune infiltration in cancer [65]. According to these data, in MS patients, it may function as an indicator of immune cell accumulation, and could be linked with NET formation. However, to our knowledge, no data are available in the literature regarding more specific MS-related functions.

The *HDC* gene encodes a key protein for histamine production, influencing chronic inflammation, neurotransmission, and immune responses [66,67]. Elevated *HDC* expression may be indicative of active immune cell recruitment, a phenomenon more consistently observable in SPMS compared to RRMS [68]. Histamine’s regulatory role in MS models further supports this observation [69]. However, *HDC* overexpression’s significance should be carefully evaluated due to histamine’s complex effects on immune cells through various receptors [67,69]. Our results suggest that *HDC* overexpression is a potential marker for MS progression, but further studies are needed to precisely assess this finding given the diverse effects of histamine in MS.

The *ITGB5* gene encodes a beta subunit of integrin, which can combine with several alpha chains to form multiple types of integrin heterodimers. While no specific studies have investigated ITGB5’s role in MS, data from the literature indicate a positive correlation of ITGB5 with immune infiltration in cancer [70]. We could hypothesize that immune infiltration may also occur in MS lesions; thus, *ITGB5* could prove to be a useful indicator of the active progression of MS.

The *LPAR5* gene encodes for a protein implicated in the mechanism of immune tolerance [71]. LPAR5 is an important regulator of T-cell activation [72]. Increased LPAR5 expression may serve as a feedback mechanism in activated T-cells, enhancing responsiveness to inhibitory signals. We can hypothesize that this up-regulation could be related to negative feedback due to decreased ligand (LPA) production. Interestingly, data from the literature evidence that LPA production is impaired/diminished in MS’s active phase and increased in symptom-free intervals [73], supporting this hypothesis. A chronic low concentration of LPAR5 may be one of the triggers for the shift of MS to its progressive form, the SPMS subtype, which does not have remitting intervals; thus, it may be a useful marker of the SPMS transition.

The *MICB* gene encodes for a protein that, through its association with the NKG2D receptor, can stimulate immune cells, potentially leading to autoreactive T-cell stimulation [74]. Several studies have investigated its possible correlation with MS and highlighted its significant association with the disease [75,76]. Interestingly, it has been observed that this protein reaches higher concentrations in MS, with the highest levels being observed during active relapses [77]. It can be hypothesized that in SPMS, where there are no relapse time frames, *MICB* expression is continuously stimulated, leading to the up-regulation we detected in our data.

The *PTPRC* gene encodes for a protein abundantly expressed in leukocytes, which is involved in the antigen–T-cell receptor/CD3/CD45 signal transduction [78]. Several investigations have been performed to evaluate its potential association with autoimmune diseases. According to these investigations, PTPRC alterations may increase the overall reactivity of the immune system and thus can predispose an individual to many different types of autoimmune diseases, including MS [79,80]. The prolonged immune activation promoted by PTPRC may be linked with the progressive phase of MS, thus nominating this gene as a potential biomarker.

The *S100A6* gene is expressed in a wide range of cell types, and appears to mainly contribute to cellular Ca^2+^ signaling and play a role in stress responses in brain [81]. Although its biological activity has not been completely elucidated, *S100A6* was found to be overexpressed in MS patients and has already been suggested as a potential biomarker of this disease [82,83]. Some studies suggest that *S100A6*’s increased expression may affect NFAT transcriptional activity, which has an established role in regulating the immune system [82]. Our results overall showed an up-regulation of this gene, and support the observations in the literature regarding its use as a biomarker. Additionally, our data suggest that its use may be more specific for the SPMS subtype compared to the RRMS subtype.

The *SELP* gene encodes for a protein that has a major role in myeloid cells’ adhesion to the endothelium and their infiltration into lesions [84]. Interestingly, data from the literature have associated mutations within this gene with MS [85]. Regarding our results, we found an up-regulation of *SELP* in SPMS patients. Additionally, *SELP* expression is up-regulated by histamine [86], linking its up-regulation to *HDC*, a previously discussed gene. This up-regulation may indicate an increased recruitment of autoreactive immune cells at lesion sites, exacerbating the inflammatory process and promoting MS progression. Constantly elevated SELP production could serve as an indicator of this process, suggesting it as a potential marker for SPMS.

The *SLAMF8* gene is expressed in a variety of activated myeloid cells, and regulates a variety of immune responses [87]. While no studies directly link *SLAMF8* to MS, its up-regulation has been reported in autoimmune inflammation states [88], suggesting a potential role of *SLAMF8* in immune-related diseases [89]. Up-regulated *SLAMF8* is associated with increased disease activity and inflammation in autoimmune diseases [90], and its involvement in the activation of macrophages during inflammation has been observed in cancer studies [89,91]. Our findings highlight SLAMF8’s overexpression in SPMS, potentially facilitating antigen presentation and local immune responses [89], thereby aggravating the autoimmune process and MS progression.

The *TRIM10* gene encodes for a protein involved in the IFN/JAK/STAT signaling pathway, regulating the immune response [92,93]. Increasing evidence highlights its association with autoimmune diseases [94], including MS [95]. While the exact molecular mechanisms triggered by TRIM10 remain elusive [96], it likely regulates the IFN response. Our results support a potential implication of this gene in MS, with its up-regulation being linked with the SPMS subtype.

The *WWOX* gene encodes for a tumor suppressor, mainly correlating its function to cancer [97], but it is also associated with the CNS’s development and function [98], as well as with immune cell proliferation and maturation [99,100]. While the exact molecular cascades regulated by WWOX are still being investigated [100,101], the *WWOX* gene exhibits higher transcriptional levels in inflammatory conditions [100]. The literature suggests a potential association of *WWOX* with MS [98,102], consistent with our results. However, some studies report its down-regulation, rather than up-regulation, in chronic active MS lesions [103], warranting further investigation into WWOX’s specific involvement.

In summary, genes involved in the dysregulation of immune tolerance and activation of immune cells, coupled with the increased exposure of myelin to immune cells due to chemokine-guided recruitment, may be indicative of the transition from RRMS to SPMS. Our study also aimed to infer key processes involved in this transition. Enriched BPs specific to SPMS are reported in Table 1 and further details are given in the Supplementary Materials. Our three-layered comparison evidenced three processes: the negative regulation of the calcineurin–NFAT signaling cascade (GO:0070885), the negative regulation of calcineurin-mediated signaling (GO:0106057), and the regulation of the defense response to viruses by a host (GO:0050691). These ontologies, overall, implicate the potential role of Ca^2+^ signaling cascades in immunity. Indeed, Ca^2+^ is an essential signaling molecule that controls a wide range of biological functions. The immune system is not an exception: here, Ca^2+^ plays a central role in several functions in immune cells and inflammation [100,104]. In particular, through the calcineurin–NFAT pathway, it regulates the transcription of genes essential for innate and adaptive immunity [105]. The literature has evidenced how this pathway is dysregulated in autoimmune diseases [104]. Interestingly, Ca^2+^–calcineurin signaling may also facilitate immune cell recruitment in the brain lesions, since it may disrupt the blood–brain barrier [106]. This evidence is in line with our data. Moreover, our results suggest that dysregulation of this signaling pathway may be specifically involved with the progressive subtype of MS. However, to further elucidate the molecular mechanisms, further studies would be needed. Regarding the regulation of the defense response to viruses, it is known that abnormal functioning of the related processes may predispose an individual to autoimmunity [107]. The correlation with SPMS may be due to a more constant presence of inflammation in this condition, which elevates cytokine concentrations and results in the dysregulation of the defense response.

Some limitations are worth being mentioned. Our data were based on publicly available datasets that have little to no available clinical information data. As such, we cannot exclude the effect of possible confounders (including, but not limited to, comorbidities, previous therapies, or other concomitant treatments) in our analyses.

## 4. Materials and Methods

### 4.1. Flowchart of the Analysis

**Figure 6:**
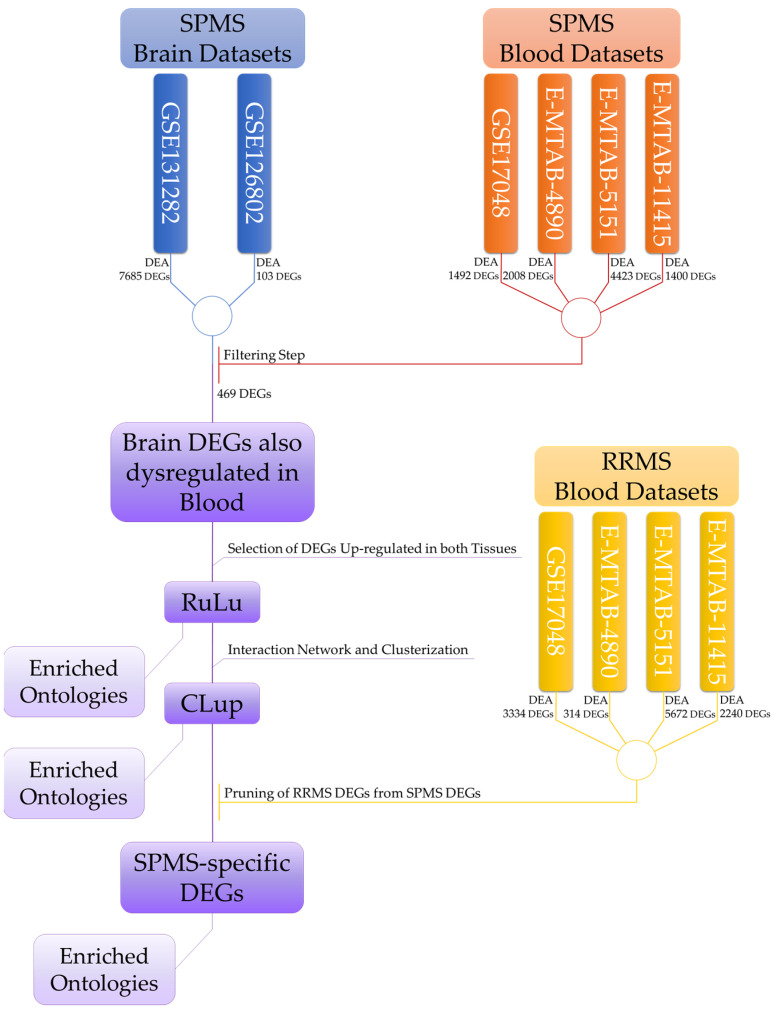
The flowchart of our analysis. The filtering step was performed by applying the cut-off described in Section 2.3. A similar cut-off was also applied for the RRMS DEGs before the pruning step. Details of the DEGs obtained in the DEA are reported in the Supplementary Materials.

The present study is a multistep analysis with the aim of highlighting potential biomarkers informative of the SPMS transition. Below, in Figure 6, we report the flow chart of our analysis.

### 4.2. Dataset Selection

A systematic research process was undertaken across publicly accessible databases Gene Expression Omnibus (GEO) [108] and ArrayExpress [109]. The chosen datasets encompassed individuals diagnosed with MS alongside HC subjects, with detailed categorization based on MS subtype. A key criterion for dataset inclusion was the presence of samples specifically representing SPMS. These samples originated from blood or brain tissues. Furthermore, each selected dataset was required to exhibit a sufficiently robust sample size to bolster the statistical robustness of our analyses. The finalized datasets enlisted for the analyses performed on the brain were GSE126802 [16] and GSE131282 [17]. The datasets selected for the blood analyses were GSE17048 [18], E-MTAB-11415 [19], E-MTAB-4890 [20], and E-MTAB-5151 [21]. The above-reported datasets included a total of 116 SPMS samples (41 from brains and 75 from blood) and 175 HCs (23 from brains and 152 from blood). Additionally, we isolated all RRMS patients used for the analysis from the four blood datasets. The number of RRMS patients present in the aforementioned datasets was 162. All of the databases were accessed on 1 December 2023 to identify appropriate datasets.

### 4.3. Dataset Description

Details on the datasets are publicly available on the GEO database and ArrayExpress database, as well as in the original submitters’ publications. Table 2 reports the descriptives of the datasets analyzed and the original submitters’ publications.

### 4.4. Annotation of Probes and Genes 

Due to the differences in terms of the dates of data collection and the instruments/technologies used between the databases, we performed two annotation steps to retrieve gene identifiers from the probes. The first step was based on the probes’ names according to the chip array used for each dataset. This step was performed automatically through a bioinformatics approach. In the second step, the results from the first step were manually checked to update the gene names. Any data displaying inconsistencies or deemed unreliable were eliminated in this step.

### 4.5. Bioinformatics Analysis

The workflow implemented in BioTEA v1.1.0 [111] was executed to acquire, preprocess, and conduct a DEA using microarray data from the selected datasets. Initially, the pipeline encompassed data retrieval from raw CEL files and data preprocessing stages. BioTEA performed the reading, parsing, background subtraction, quantile–quantile normalization, and quality checks of the raw expression data, ensuring robust computational reproducibility. DEA was conducted independently on each dataset to discover every DEG for each comparison. Rank Product (RankProd v3.28.0 package in R v4.3.3 environment) was applied for the DE analysis [112]. This selection was motivated by prior findings indicating the biological relevance of RankProd results. Moreover, RankProd is better suited for cross-dataset comparisons as it evaluates gene ranks rather than absolute expression values. In our analysis, we accepted as DEGs all genes with an adjusted *p*-value (q-value) < 0.05. The FDR method was applied to adjust the *p*-values to reduce the number of false positives. The regulation status of each DEG was determined using the original log_2_ fold change obtained from the analysis of each dataset.

The STRING database [113], integrated with Cytoscape software [114], was employed to construct interaction networks among the DEGs in our study. STRING provides a comprehensive resource for assessing protein–protein interactions, amalgamating both experimental and predicted data. The default confidence score cut-off (kappa = 0.4) was utilized for network construction, based on the STRING dataset version 12.0. In addition to a network analysis, the list of DEGs was subjected to gene ontology (GO) enrichment analysis to identify statistically significant alterations in the gene ontologies. The GSEAPY package v1.1.1 in the Python environment was utilized for the enrichment analysis.

### 4.6. Differential Expression Analysis Specifics

In the performed DEA, we considered as up-regulated all DEGs that assumed a statically confirmed increased expression in the SPMS group compared to the HC group for each dataset, while the down-regulated DEGs were those less expressed in the SPMS group compared to the HCs for each dataset. No filters based on fold changes were applied as we wanted to maintain the most comprehensive perspective possible.

## 5. Conclusions

We highlighted 21 genes possibly involved with MS and in particular with the RRMS-to-SPMS transition. According to their discussed functions, these genes may be directly involved with SPMS progression or may appear overexpressed as an attempt to counter the immune activation. These phenomena are likely more evident in the SPMS phase as the relapsing–remitting patients are still able to have free-of-symptoms windows, suggesting that the inhibitory mechanics still retain their ameliorating effect on immune activation. This balance is instead skewed towards immune activation in SPMS patients. Such observations may suggest that the primary trigger of the RRMS-to-SPMS transition is related to the disruption of the inhibitory cascades controlling immune cells’ activity and tolerance against antigen mechanisms. In this sense, a potential process involved may be related to Ca^2+^, more specifically, the calcineurin–NFAT pathway, which we found to be enriched specifically in SPMS patients. Its role in this condition, however, will need further investigations to be elucidated. Regarding their potential use in the MS clinical field, the selected genes may set up the groundwork for the definition of potential markers of the RRMS-to-SPMS transition, and could be useful for further studies and in developing new treatment designs to slow disease progression.

## Figures and Tables

**Table 1:** SPMS-specific biological processes based on the three-layered comparison.

Term	Overlap	*p*-Value	q-Value	Genes
Negative regulation of calcineurin–NFAT signaling cascade (GO:0070885)	3/11	5.32 × 10^−5^	3.51 × 10^−2^	*HOMER2*; *FHL2*; *HOMER3*
Negative regulation of calcineurin-mediated signaling (GO:0106057)	3/11	5.32 × 10^−5^	3.51 × 10^−2^	*HOMER2*; *FHL2*; *HOMER3*
Regulation of defense response to virus by host (GO:0050691)	4/37	1.27 × 10^−4^	4.19 × 10^−2^	*DHX9*; *CGAS*; *TARBP2*; *MICB*

In table, we report the biological processes (BPs) significantly enriched from the genes with different expression patterns observed in SPMS and RRMS. From the list of BPs, we filtered out the processes that were significantly enriched from the genes with the same expression patterns observed in SPMS and RRMS.

**Table 2:** Descriptives of the samples under investigation.

Dataset	Tissue	Secondary Progressive (SPMS)			Relapsing–Remitting (RRMS)			Healthy Controls (HCs)			References
		*n*	% Females	Mean Age	*n*	% Females	Mean Age	*n*	% Females	Mean Age	
GSE126802	Brain	18	100.0%	61.3 ± 13.4	-	-	-	9	100.0%	61.7 ± 10.1	[16]
GSE131282	Brain	23	73.9%	75.1 ± 14.8	-	-	-	14	28.6%	58.5 ± 14.5	[17]
GSE17048	Blood	20	75.0%	57.5 ± 9.8	36	80.6%	48.5 ± 9.0	45	64.4%	48.5 ± 13.5	[18,110]
E-MTAB-4890	Blood	21	52.4%	54.3 ± 11.1	52	61.5%	37.4 ± 10.2	40	50.0%	33.3 ± 10.4	[20]
E-MTAB-5151	Blood	13	69.2%	41.8 ± 9.3	21	57.1%	41.2 ± 8.1	27	48.2%	42.4 ± 9.1	[21]
E-MTAB-11415	Blood	21	47.6%	54.4 ± 11.1	53	60.4%	37.4 ± 10.1	40	50.0%	33.4 ± 10.5	[19]

In table, we report for each dataset the number of subjects (*n*), the percentage of females, and the mean age for each group (SPMS, RRMS, and HCs) used in our investigation. The original submitters’ publications are also indicated in the References column.

## Data Availability

The data presented in this study are openly available in the Gene Expression Omnibus (GEO) database (https://www.ncbi.nlm.nih.gov/gds; last accessed on 1 December 2023), under the accession numbers GSE126802, GSE131282, and GSE17048, respectively; as well as in the Expression Atlas database (https://www.ebi.ac.uk/gxa/home; last accessed on 1 December 2023) under the accession numbers E-MTAB-4890, E-MTAB-5151, and E-MTAB-11415, respectively.

## Supplementary Materials

The following supporting information can be downloaded at https://www.mdpi.com/article/10.3390/ijms25063374/s1.
