# Supplementary material for: In Silico Analysis Highlights Potential Predictive Indicators Associated with Secondary Progressive Multiple Sclerosis
Source: Int J Mol Sci. 2024 Mar 16;25(6):3374. doi: 10.3390/ijms25063374 (PMC10970138; doi:10.3390/ijms25063374)
Supplement: Supplementary file 1 [file ijms-25-03374-s001.zip › Supplementary Figure S1.pdf]

Supplementary Figure S1

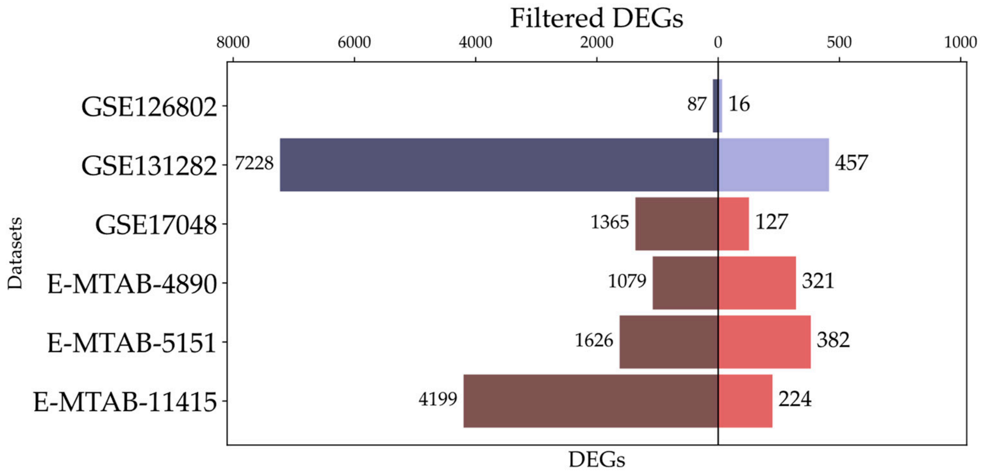

Supplementary Figure S1. In the plot we summarize the total DEGs we obtained from each dataset and highlighted the number of DEGs that survived the filtering step (on right-hand side). In purple we report the DEGs from brain datasets while in red we report the DEGs from blood datasets. The figure is complemented with supplementary Table 1 data, in which we report the the survived DEGs names, their fold change and pValues.
