# Supplementary material for: In Silico Analysis Highlights Potential Predictive Indicators Associated with Secondary Progressive Multiple Sclerosis
Source: Int J Mol Sci. 2024 Mar 16;25(6):3374. doi: 10.3390/ijms25063374 (PMC10970138; doi:10.3390/ijms25063374)
Supplement: Supplementary file 1 [file ijms-25-03374-s001.zip › Supplementary Figure S2.pdf]

Supplementary Figure S2

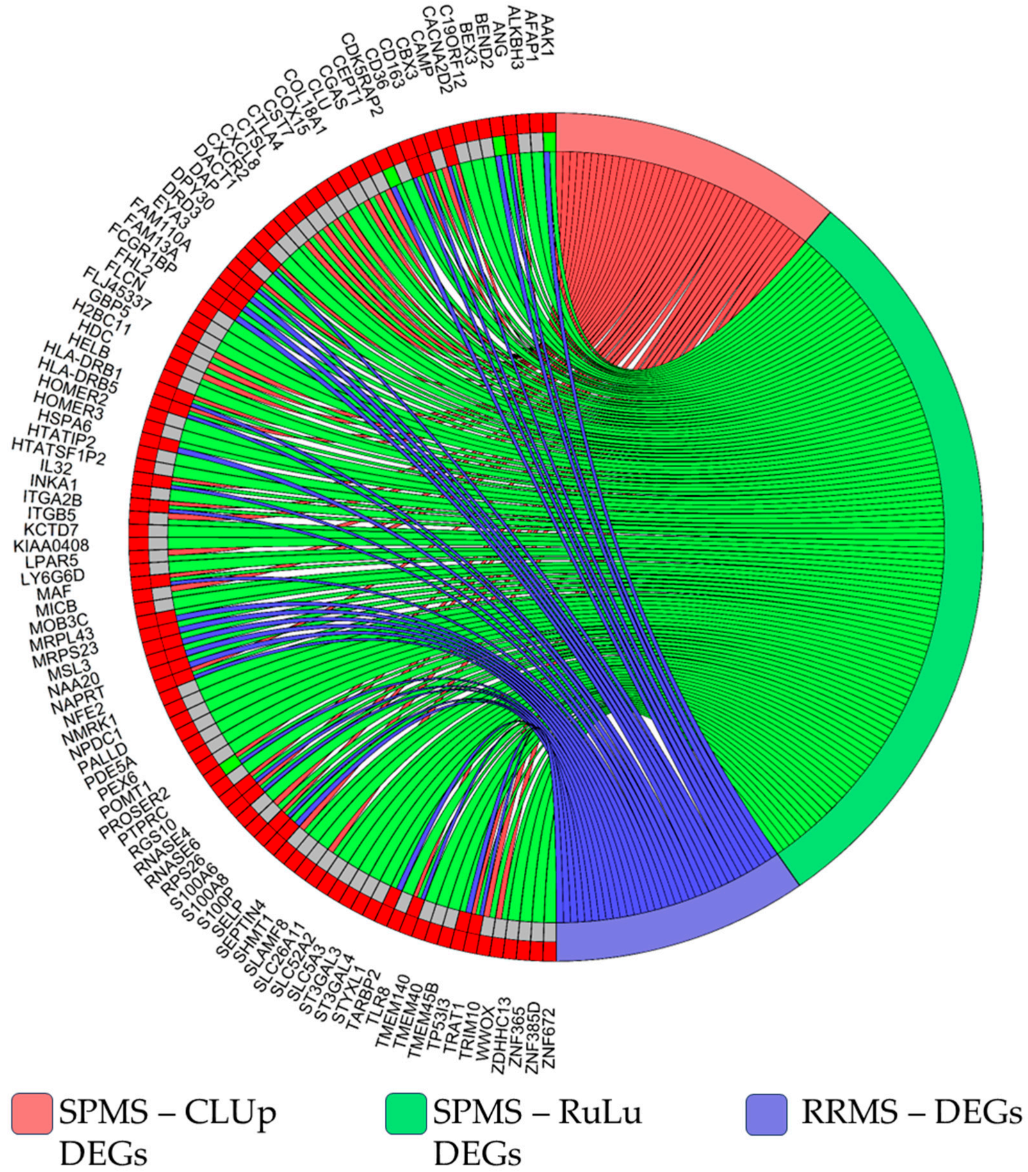

Supplementary Figure S2. In the chord plot we report RuLu DEGs (left-hand side) and the phenotypic groups in which they were differentially expressed (right-hand side). On the left, the outer ring reports the expression status in SPMS patients, while the inner one reports the expression status in RRMS patients. Red genes are up-regulated and Green down-regulated, while Gray represent no Differential expression. DEGs belonging to CLUp are linked to the red sector on the right-hand side, DEGs belonging to RuLu are linked to the green sector and genes found to also be differentially expressed in RRMS patients are linked to the blue sector.
